# In the March 2013 issue

**DOI:** 10.1590/S1980-57642013DN70100001

**Published:** 2013

**Authors:** Paulo Caramelli, Facundo Manes, Thomas Bak

**Affiliations:** Guest Editors

Frontotemporal Lobar Degeneration (FTLD) is the second most frequent cause of early-onset dementia, which includes three major clinical subtypes, namely behavioral variant frontotemporal dementia, primary progressive aphasia and semantic dementia. These dementia syndromes share as a common feature the presence of focal atrophy affecting the anterior parts of the frontal or temporal lobes, presenting clinically as progressive impairment in social function, personality changes, executive dysfunction or a decline in language abilities. Moreover, they may also overlap with motor neuron disease or with extrapyramidal disorders, such as corticobasal degeneration and progressive supranuclear palsy.

The FTLD syndromes represent a major challenge for the clinician, both in terms of diagnosis and therapeutic management, and even more for the patients and their families, who have to cope with very distressing symptoms.

Considerable research efforts have been made in the last two decades in order to unravel the clinical, genetic and pathological basis of FTLD syndromes. These advances are crucial for the development of new diagnostic tools, which may allow early and more specific detection of these disorders, and will also help to build up and test new therapies for such devastating conditions.

This issue of **Dementia & Neuropsychologia** publishes reviews, original works and case reports on FTLD by prominent researchers from Argentina, Australia, Brazil, Chile, France, India, Peru, United Kingdom and United States of America, who have accepted our invitation to submit their studies for consideration by the journal. These manuscripts represent a significant contribution to the field and might be of great interest to clinicians and researchers.

We express our deep gratitude to all authors who have contributed to this issue, coming from four continents, thus making this initiative truly international. We also thank Prof. Ricardo Nitrini, editor of **Dementia & Neuropsychologia**, for trusting in our competence to edit this issue, and to Mrs. Alair Mariana dos Santos Silva, for her dedicated secretarial assistance.

Paulo Caramelli Guest EditorFacundo Manes Guest EditorThomas Bak Guest Editor

